# Dietary score and the risk of oral cancer: a case-control study in southeast China

**DOI:** 10.18632/oncotarget.16659

**Published:** 2017-03-29

**Authors:** Fa Chen, Lingjun Yan, Lisong Lin, Fengqiong Liu, Yu Qiu, Jing Wang, Junfeng Wu, Fangping Liu, Jiangfeng Huang, Lin Cai, Baochang He

**Affiliations:** ^1^ Department of Epidemiology and Health Statistics, School of Public Health, Fujian Medical University, Fujian, China; ^2^ Key Laboratory of Ministry of Education for Gastrointestinal Cancer, Fujian Medical University, Fujian, China; ^3^ Fujian Key Laboratory of Tumor Microbiology, Fujian Medical University, Fuzhou, China; ^4^ Department of Oral and Maxillofacial Surgery, The First Affiliated Hospital of Fujian Medical University, Fujian, China; ^5^ Laboratory of Facial Plastic and Reconstruction of Fujian Medical University, Fujian, China; ^6^ Laboratory Center, School of Public Health, Fujian Medical University, Fujian, China

**Keywords:** oral cancer, dietary score, tobacco smoking, alcohol drinking, case-control study

## Abstract

This study aims to develop a simple dietary score to comprehensively evaluate the role of diet in the risk of oral cancer. A case-control study including 930 oral cancer cases and 2667 frequency-matched controls was performed in Fujian, China. Unconditional logistic regression model was used to estimate the effects of dietary factors on oral cancer. After adjustment for potential confounders, less intake of domestic meat (< 3 times per week), fish (< 3 times per week), seafood (< 3 times per week), leafy vegetables (< 1 time per day), other vegetables (< 1 time per day), fruits (< 3 times per week), milk and dairy products (< 1 time per week) and eggs (< 5 times per week) were significant risk factors for oral cancer. Then these variables were incorporated to establish dietary risk score. Assessed by the receiver operating characteristic curve, the score showed a satisfactory discriminatory capacity, with an area under the curve of 0.682 (95% CI: 0.662–0.702). Moreover, the score was positively associated with the risk of oral cancer as quartiles, and the association was apparently stronger in tobacco smokers or alcohol drinkers. Additionally, there were significant multiplicative interactions between the score and tobacco smoking or alcohol drinking for oral cancer. In the present study, a convenient dietary score with satisfactory discriminatory capacity was developed to assess the collected effect of dietary factors on oral cancer, which could provide a new strategy for the prevention of oral cancer through changing in dietary habits.

## INTRODUCTION

Oral cancer is the eighth most common cancer in the world, with approximately 90% of cases being squamous cell carcinoma (SCC) [1, 2]. In China, it has been reported that there are 21,413 new cases and 11,333 deaths of oral cancer in 2012 [3]. Given that the disfigurement consequence and social communication difficulties after treatment and a permanent reduction in quality of life, therefore, much attention should be paid to the prevention of oral cancer.

Although tobacco smoking and alcohol drinking are known to be main risk factors for oral cancer [4, 5], diet also plays an important role in the etiology of oral cancer. It was well established that high consumption of fresh vegetables, fruits, fish and seafood could protect against oral cancer [6–8]. Several epidemiological studies indicated that high intake of red and processed meat were associated with increased risk of oral cancer [9, 10]. Whereas, there is limited epidemiologic data on the collective effect of diet factors on oral cancer risk.

Since the diet contains a variety of food, diet-based score system which takes overall diet food into account becomes a promising method for comprehensively assessing the association between diet and oral cancer risk. A recent study in Italy established a Mediterranean diet score (MDS) based on nine traditional Mediterranean diet foods for oral and pharyngeal cancer [11]. However, so far, few studies have developed a convenient and efficient scoring system according to Chinese dietary patterns for oral cancer.

Therefore, we performed a case-control study with large samples size to create a dietary score for comprehensively estimating the role of diet on the risk of oral cancer and identifying high-risk individuals for oral cancer in southeast of China.

## RESULTS

The main characteristics of 930 oral cancer patients and 2667 controls are showed in Table 1. Cases and controls had similar distributions of age, gender, education level and residence (*P* > 0.05). BMI and family history of cancer were significantly different between two groups, and cases were more likely to have tobacco smoking and alcohol consumption than controls (*P <* 0.05). With regard to histological types, the majority of patients (80.22%) were oral squamous cell carcinoma, whereas the proportions of oral adenocarcinoma (OA) and other types were only 11.94% and 7.84%, respectively.

**Table 1:** Main characteristics of case and control subjects

Variables	Cases (%) (*n* = 930)	Controls (%) (*n* = 2667)	χ^2^	*P*-value
Age (years)			0.017	0.897
≤ 60	510 (54.84)	1456 (54.59)		
> 60	420 (45.16)	1211 (45.41)		
Gender			0.003	0.955
Male	588 (63.23)	1689 (63.33)		
Female	342 (36.77)	978 (36.67)		
Education level			5.117	0.077
Illiterate	108 (11.61)	313 (11.74)		
Primary and middle school	579 (62.26)	1557 (58.38)		
High school and above	243 (26.13)	797 (29.88)		
Residence			3.475	0.062
Rural	523 (56.24)	1593 (59.73)		
Urban	407 (43.76)	1074 (40.27)		
BMI (kg/m^2^)^a^			112.477	< 0.001
18.5–23.9	591 (63.55)	1499 (56.20)		
< 18.5	125 (13.44)	145 (5.44)		
≥ 24	214 (23.01)	1023 (38.36)		
Family history of cancer			18.726	< 0.001
No	774 (83.23)	2366 (88.71)		
Yes	156 (16.77)	301 (11.29)		
Tobacco smoking			81.419	< 0.001
No	482 (51.83)	1822 (68.32)		
Yes	448 (48.17)	845 (31.68)		
Alcohol consumption			74.460	< 0.001
No	603 (64.84)	2107 (79.00)		
Yes	327 (35.16)	560 (21.00)		
Denture wearing			34.302	< 0.001
No	481 (51.72)	1671 (62.65)		
Yes	449 (48.28)	996 (37.35)		
Recurrent oral ulceration			100.673	< 0.001
No	836 (89.89)	2605 (97.68)		
Yes	94 (10.11)	62 (2.32)		

Table 2 presents the daily food intake and the risk of oral cancer, and risk scores for the final predictors. After adjustment for potential confounders, less intake of domestic meat (< 3 times per week), fish (< 3 times per week), seafood (< 3 times per week), leafy vegetables (< 1 time per day), other vegetables (< 1 time per day), fruits (< 3 times per week), milk and dairy products (< 1 time per week) and eggs (< 5 times per week) were significantly associated with increased risk of oral cancer. Moreover, compared to subjects who consumed pickled food < 1 time per week, those who consumed pickled food more than once per week were more susceptible to oral cancer (OR = 1.21, 95% CI: 1.02–1.43). Then, risk scores for these variables were calculated according to β coefficient. Since red meat, processed meat and beans and soy products showed no significant associations with oral cancer risk through multivariable-adjusted model, these three variables are not incorporated into the final scoring model.

**Table 2:** Daily food intake and the risk of oral cancer and risk scores for the final predictors

Variables	Cases (%)*n* = 930	Controls (%)*n* = 2667	Unadjusted*OR* (95% *CI*)	Adjusted*OR* (95% *CI*)^a^	Risk score
Poultry meat (per week)					
≥ 3 times	130 (13.98)	615 (23.06)	1.00	1.00	0
< 3 times	800 (86.02)	2052 (76.94)	1.84 (1.50–2.27)	1.71 (1.37–2.12)	5
Fish (per week)					
≥ 3 times	430 (46.24)	1590 (59.62)	1.00	1.00	0
< 3 times	500 (53.76)	1077 (40.38)	1.72 (1.48–2.00)	1.75 (1.49–2.05)	6
Seafood (per week)					
≥ 3 times	206 (22.15)	937 (35.13)	1.00	1.00	0
< 3 times	724 (77.85)	1730 (64.87)	1.90 (1.60–2.27)	1.99 (1.65–2.40)	7
Leafy Vegetables (per day)					
≥ 1 time	786 (84.52)	2552 (95.69)	1.00	1.00	0
< 1 time	144 (15.48)	115 (4.31)	4.07 (3.14–5.26)	3.91 (2.98–5.14)	14
Other vegetables (per day)					
≥ 1 time	755 (81.18)	2489 (93.33)	1.00	1.00	0
< 1 time	175 (18.82)	178 (6.67)	3.24 (2.59–4.05)	3.16 (2.49–4.01)	12
Fruits (per week)					
≥ 3 times	321 (34.52)	1651 (61.90)	1.00	1.00	0
< 3 times	609 (65.48)	1016 (38.10)	3.08 (2.64–3.60)	3.00 (2.53–3.56)	11
Milk and dairy products (per week)					
≥ 1 time	326 (35.05)	1243 (46.61)	1.00	1.00	0
< 1 time	604 (64.95)	1424 (53.39)	1.62 (1.39–1.89)	1.60 (1.36–1.90)	5
Eggs (per week)					
≥ 5 times	270 (29.03)	1012 (37.95)	1.00	1.00	0
< 5 times	660 (70.97)	1655 (62.05)	1.49 (1.27–1.76)	1.44 (1.21–1.71)	4
Pickled food (per week)					
< 1 time	639 (68.71)	1932 (72.44)	1.00	1.00	0
≥ 1 time	291 (31.29)	735 (27.56)	1.20 (1.02–1.41)	1.21 (1.02–1.43)	2
Red meat (per week)					
< 5 times	433 (46.56)	1267 (47.51)	1.00	1.00	
≥ 5 times	497 (53.44)	1400 (52.49)	1.04 (0.89–1.21)	1.01 (0.86–1.19)	
Processed meat (per week)					
< 1 time	801 (86.13)	2284 (85.64)	1.00	1.00	
≥ 1 time	129 (13.87)	383 (14.36)	0.96 (0.77–1.19)	1.03 (0.82–1.29)	
Beans and soy products (per week)					
≥ 1 time	624 (67.10)	1824 (68.39)	1.00	1.00	
< 1 time	306 (32.90)	843 (31.61)	1.06 (0.90–1.24)	1.12 (0.95–1.33)	

^a^adjusted for age, gender, education, residence, BMI, family history of cancer, tobacco smoking, alcohol consumption, denture wearing, recurrent oral ulceration.

**Figure 1:**
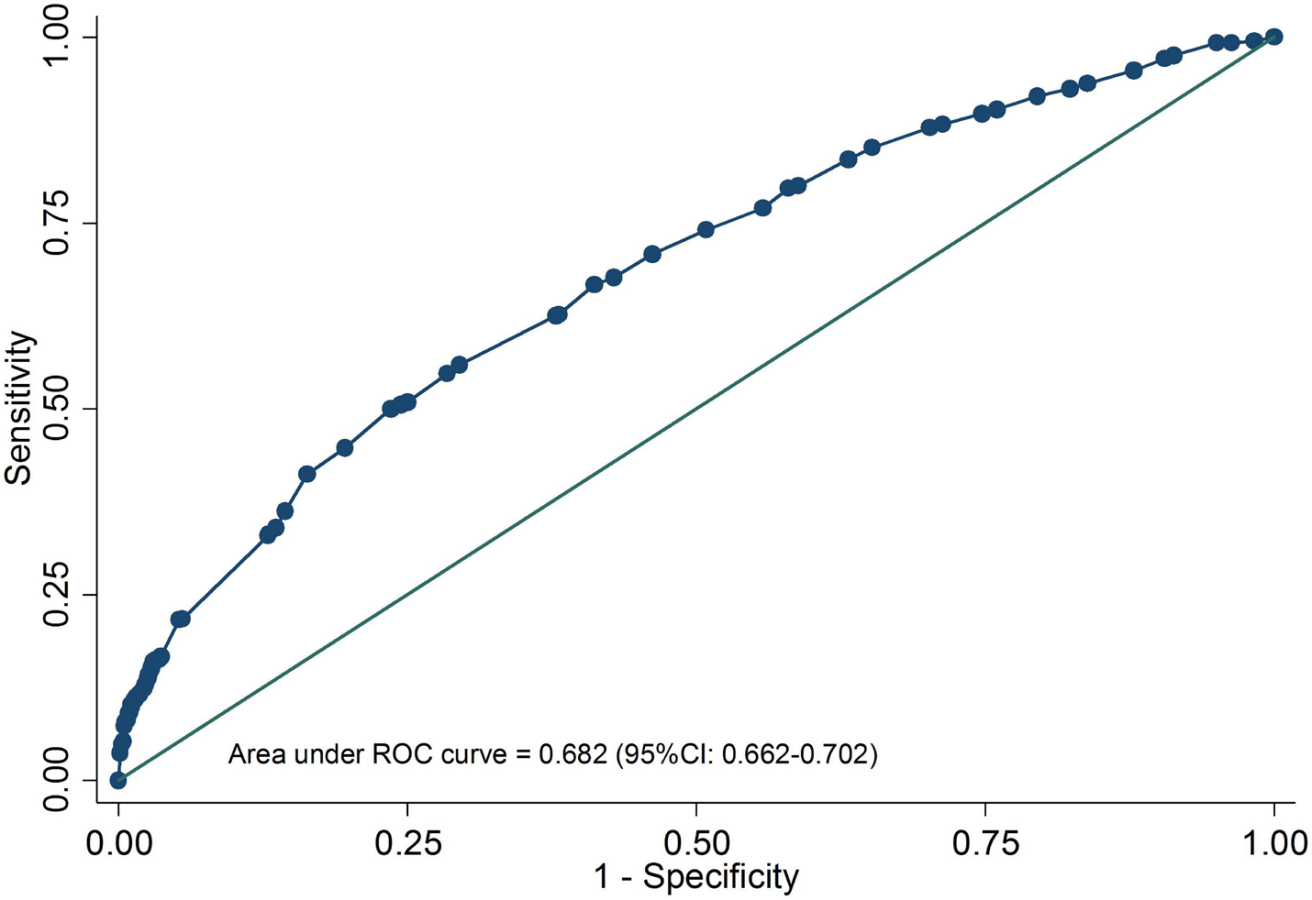
Receiver operating characteristic curve of dietary score for predicting oral cancer risk

As shown in Table 3, the dietary risk score ranged from 0 to 66. The medians (quartiles) of case and control group were 31.5 (21, 38) and 22(13, 30), respectively. The cases had significantly higher dietary risk score than controls (*P <* 0.05). A receiver operating characteristic (ROC) curve was plotted to assess the predictive accuracy of dietary risk score, with area under the curve of 0.682 (95%CI: 0.662–0.702, Figure 1). And then the dietary score was categorized into four groups based on the quartiles of controls. The higher the score, the greater the risk of oral cancer (*P* for trend < 0.001). Compared with subjects in the lowest risk group, those in the highest risk group had a nearly 5-fold increased risk of oral cancer (OR = 5.10, 95%CI: 3.90–6.67). Similar result was observed in OSCC patients. But only quartile 4 of the score showed a positive relationship with OA and others. Moreover, there was no significant association in case-case comparisons.

**Table 3:** Associations between quartiles of dietary score and oral cancer risk

Quartiles of dietary score	Range of score	Case–control comparisons							Case–case comparisons
		Controls (*n* = 2667)	All patients		OSCC		OA and others		OSCC VS OA and others
			*n* = 930	*OR* (95% *CI*)^a^	*n* = 746	*OR* (95% *CI*)^a^	*n* = 184	*OR* (95% *CI*)^a^	*OR* (95% *CI*)^a^
Quartile 1	0–12	640	90	1.00	63	1.00	27	1.00	1.00
Quartile 2	13–21	671	151	1.57 (1.17–2.10)	123	1.84 (1.31–2.59)	28	1.00 (0.58–1.72)	1.63 (0.86–3.08)
Quartile 3	22–29	689	215	2.14 (1.61–2.85)	173	2.50 (1.81–3.47)	42	1.41 (0.85–2.33)	1.50 (0.83–2.72)
Quartile 4	30–66	667	474	5.10 (3.90–6.67)	387	6.08 (4.45–8.30)	87	3.27 (2.06–5.19)	1.59 (0.92–2.73)
*P* for trend				< 0.001		< 0.001		< 0.001	0.029

Abbreviations: OSCC, oral squamous cell carcinoma; OA, oral adenocarcinoma.

^a^adjusted for age(y), gender, education, residence, BMI, family history of cancer, tobacco smoking, alcohol consumption, denture wearing, recurrent oral ulceration.

The associations between quartiles of dietary score and oral cancer risk stratified by tobacco smoking and alcohol drinking are shown in Table 4. There appeared to be a gradient increased risk in smokers as well as non-smokers, and similar results were also observed in alcohol drinkers and non-alcohol drinkers (all *P* for trend < 0.001). Whereas, the ORs were greater among smokers or alcohol drinkers when compared with non-smokers or non-alcohol drinkers, respectively. Additionally, we found significant multiplicative interactions between dietary score and tobacco smoking or alcohol drinking for oral cancer, the ORs_multiplicative_ were 1.67 (95%CI: 1.54–1.82, *P <* 0.001) and 1.49 (95% CI: 1.38–1.62, *P <* 0.001), respectively.

**Table 4:** Quartiles of dietary score and risk of oral cancer, stratified by tobacco smoking and alcohol drinking status

Variables	Quartiles of dietary risk score				*P* for trend	*P* for interaction	*OR*_multiplicative_ (95% *CI*)
	Quartile 1	Quartile 2	Quartile 3	Quartile 4			
Smoker						< 0.001	1.67(1.54–1.82)^b^
Cases/ Controls	28/166	55/187	100/222	265/270			
*OR* (95%*CI*)^a^	1.00	1.81 (1.07–3.06)	2.96 (1.81–4.85)	7.32 (4.59–11.68)	< 0.001		
Non-smoker							
Cases/ Controls	62/474	96/484	115/467	209/397			
*OR* (95%*CI*)^a^	1.00	1.48 (1.03–2.12)	1.88 (1.32–2.68)	4.16 (2.97–5.83)	< 0.001		
Alcohol drinker						< 0.001	1.49 (1.38–1.62)^c^
Cases/ Controls	19/98	44/142	76/139	188/181			
*OR* (95%*CI*)^a^	1.00	1.90 (1.02–3.54)	3.23 (1.78–5.86)	6.93 3.92–12.26)	< 0.001		
Non-alcohol drinker							
Cases/ Controls	71/542	107/529	139/550	286/486			
*OR* (95%*CI*)^a^	1.00	1.50 (1.07–2.09)	2.01 (1.46–2.78)	5.08 (3.75–6.89)	< 0.001		

^a^adjusted for age (y), gender, education, residence, BMI, family history of cancer, tobacco smoking, alcohol consumption, denture wearing, recurrent oral ulceration.

^b^adjusted for age (y), gender, education, residence, BMI, family history of cancer, alcohol consumption, denture wearing, recurrent oral ulceration.

^c^adjusted for age (y), gender, education, residence, BMI, family history of cancer, tobacco smoking, denture wearing, recurrent oral ulceration.

## DISCUSSION

To our knowledge, this large-scale case-control study was the first to develop a dietary score based on Chinese dietary patterns for oral cancer in southeast of China. With the increasing of dietary score, the increased risk of oral cancer was observed. Moreover, the risk was apparently greater in tobacco smokers or alcohol drinkers. Additionally, significant multiplicative interactions between dietary score and tobacco smoking or alcohol drinking were also found.

The anti-cancer effects of fresh vegetables and fruits on oral cancer have been confirmed by some previous studies [12, 13], which may attributed to their contents of carotenoids, vitamin A, vitamin C, folic acid, flavonoids and other antioxidants [14]. Our study also observed inverse relationships between fish and seafood intake and oral cancer risk, which was similar to a recent pooled analysis [7]. Fish and seafood contain polyunsaturated fatty acids, mineral salts and proteins which could inhibit tumor progression through their anti-inflammatory effects and inhibition of oxygen free radicals [15]. Supporting our findings, a recent study revealed that milk and dairy products consumption have a favorable influence on oral cancer [16], however, an opposite result was found by Bravi *et al*. [17]. Regarding to other dietary items such as eggs and pickled food on oral cancer risk, there were also inconsistent findings, with several studies suggesting direct, inverse even null relationships.

At present, accumulating studies have focused on the association between overall dietary patterns rather than single food item and risk of several tumors through establishing diet-associated score. Filomeno *et al*. [11] found that the Mediterranean diet appears to be associated with a reduced risk of oral and pharyngeal cancer using three different diet scores. Shivappa *et al*. [18] developed a dietary inflammatory index to estimate inflammatory potential of diet on risk of laryngeal cancer. Unlike these previous diet-related score, the dietary score established in this study can be calculated in a simple way and widely available with a satisfactory discriminatory capacity. It may serve as a valuable tool for estimating the overall effect of diet by integrating multiple dietary factors into a unitary score that can improve statistical efficiency.

Additionally, our data showed that the association between dietary score and the risk of oral cancer was modified by tobacco smoking and alcohol consumption, with multiplicative interactions between them. The potential mechanism might be that tobacco smoking and alcohol drinking interfered with the metabolism of some micronutrients such as alpha-carotene, beta-carotene and folate, resulting in low serum concentrations of them [19, 20]. Moreover, toxic components of tobacco and alcohol might strengthen the carcinogenesis of N-nitroso compounds contained in pickled food, and exerted a synergic effect on oral cancer risk [21].

There were several limitations in our study. Firstly, as a retrospective case-control study, recall bias should be concerned. However, all patients were new diagnosed and all participants were investigated the dietary habits one year prior to cancer diagnosis or interview (for controls), which might minimize the possibility of recall bias. Secondly, our analyses only adjusted for main confounding factors of oral cancer. Therefore, other confounders such as total energy of subjects should be included in the future studies. Thirdly, the score was established according to the frequency of food. Hopefully, dietary score based on the quantity of food intake was developed in the future studies.

In conclusion, this study successfully developed a dietary score based on nine Chinese traditional dietary items to evaluate the collected effect of dietary factors on oral cancer. This dietary score might be a useful and convenient tools for the prevention of oral cancer with satisfactory discriminatory capacity. Prospective studies are warranted to validate our findings.

## MATERIALS AND METHODS

### Study participants

We conducted a case–control study on oral cancer between September 2010 and December 2016 in Fujian province, China. 930 oral cancer patients were recruited form the First Affiliated Hospital of Fujian Medical University. As described previously [22], cases were eligible if they met the following inclusion criteria: (1) all cases were primary oral cancer patients with newly diagnosed and histologically confirmed; (2) all cases are Chinese Han population who reside in Fujian Province; (3) all cases aged 20 to 80 years. The exclusion criteria of cases included those with second primary oral cancer, recurrent or metastasized oral cancer, and previous radiotherapy or chemotherapy. 2667 controls were randomly selected from medical examination center in the same hospitals and community population, without a diagnosis of cancer or other diseases that may lead to significant changes in diet. Those who had a previous history of any malignant disease were excluded. All controls were frequency-matched to cases by age (±3 years) and gender. The recruiting rates were 98.6% for cases and 92.8% for controls. This study was approved by the Institutional Review Board of Fujian Medical University (Fuzhou, China) and an informed consent was obtained from all participants.

### Data collection

A structured questionnaire was used to collect information through face to face interviews conducted by well-trained interviewers. The questionnaire included socio-demographic characteristics, dietary data, tobacco smoking and alcohol drinking status, oral hygiene, family history of cancer and other disease.

Subjects who had smoked at least 100 cigarettes during their lifetime were considered tobacco smokers. Alcohol drinker was defined as consumed at least one drink per week and lasting for more than 6 months continuously. As for dietary data, the questionnaire contained 12 broad categories (red meat: pork/beef/lamb; domestic meat: chicken/duck/goose; processed meat: sausage/bacon/ham; fish; seafood: shrimp/crab/shellfish; milk and dairy products; eggs: chicken and duck eggs; leafy vegetables and other vegetables; fruits; beans and soy products; pickled food). Then, participants were asked how often intake of each food item according to the following options: 3 times per day; 2 times per day; 1 time per day; 5–6 times per week, 3–4 times per week; 1–2 times per week; less than 1 time per week or not at all. And these questions about dietary habits were directed one year prior to cancer diagnosis or interview (for controls).

### Statistical analysis

The distribution of main characteristics between case and control groups was analyzed using Pearson's chi-square test. Each dietary item was grouped according to the median of the controls. Unconditional logistic regression model was used to estimate the odds ratios (ORs) and corresponding 95% confidence intervals (95% CI) for the effects of dietary factors on oral cancer risk. The statistically significant variables of dietary obtained from multivariable-adjusted model were given a risk score which was equal to β coefficient multiplied by 10 and rounded to the closest integer [23]. Individually dietary score was calculated by summing the risk score of each variable mentioned above, and the ROC was plotted to assess its predictive accuracy. The difference in the score between cases and controls was compared by the Wilcoxon rank sum test. We then categorized dietary score into four groups based on the quartiles of controls for further analysis, with the lowest quartile defined as the referent category. Additionally, the potential modification effects of tobacco smoking and alcohol drinking on dietary score for oral cancer were assessed by stratified and interaction analyses with unconditional logistic regression models. Statistical significance was considered to be *P <* 0.05. All analyses were performed using R software version 3.1.1.
